# The Chemists' Exhibition, 1901

**Published:** 1901-08-31

**Authors:** 


### The Chemists' Exhibition, 1901.

This annual exhibition, which is held this week at Covent Garden Theatre, London, W.C., is the seventh of the series, and like its predecessors, contains much of interest to medical men and nurses, many of whom attended upon the opening day, August 26. Prominent among the exhibits of drugs and pharmaceutical preparations are the stalls of Messrs. Parke, Davis and Co., their Euthymol toilet preparations, Creosoted Cod-Liver oil emulsion and effervescent Lithia Tablets, being of special interest; The Wm. S. Merrell Chemical Company, where Salicylate Sodium, Hydrastis preparations and Idozen, an iodine derivative of Methyl Salicylate, were noticeable; Messrs. F. Newbery and Sons displayed Messrs. Warner's Bromo-soda, Eff. Lithia tablets, and their Eff. Kissingen and Vichy tablets, attention being called especially to the solubility of their sugar-coated pills. Messrs. Wyleys, Limited, Coventry, showed their Effervescent Tropels and Syrup Glycerophosphates, their capsicum in handy-sized tubes also being of interest. The exhibit of Messrs. Christy and Co. should specially interest dispensers, their cachets and celluloid labels for shelf ware being particularly interesting. Messrs. Hearon, Squire and Francis, Limited, had a display of medicated soaps, petroleum and cod liver oil emulsions, bougies, pessaries, and suppositories; attention being also specially called to their Gelatine Soluble Perles. The stall of Messrs. Edgars, Limited, who showed their lotion, was tastefully arranged, and attracted much attention, as did also that of Messrs. Price's Patent Candle Company, Limited, who displayed their medicated soaps and glycerol of malt preparations. Other exhibitors of soaps and toilet preparations were Messrs. E. Cook and Co., Limited, who gave special prominence to their "Riviera" soap which is superfatted and free from colouring matter; their well-known medicated soaps being also shown. Messrs. Gosnell and Co. showed their well-known "Cherry Blossom" and "Famora" toilet preparations and perfumes, their stall attracting many nurses, as also did that of Mme. Blanche Leigh, whose manufactures are rapidly extending in favour.

Foods were represented by The British Somatose Company, Limited, who displayed Somatose in its well-known forms. Messrs. Cosenza and Co. exhibited their Maggi specialities, including Maggi's Consommé and Tonic Bouillon, which is an excellent tonic and food combined. Virol, Limited, had a prominent display of their celebrated Food, and Cheltine Foods, Limited, appeared to be experiencing a brisk demand for samples of their specialities. Surgical and medical bandages, plasters, appliances, etc., were shown by Messrs. A. de St. Dalmas and Co., of Leicester, who invited attention to their Varico-leg bandage, which is claimed to be cooler than an elastic stocking and lighter in weight. Their Surgeon's Emergency case, which can be had containing either holland or flesh-coloured cambric ribbon bandages, was also worthy of note.

Mineral waters were exhibited by Messrs. Ingram and Royle, Limited, who showed the well-known Vichy and Hunyadi waters, and their Carlsbad natural mineral waters and salts. Messrs. Farrow and Jackson showed their mineral water machines which they recommend as effecting much economy in institutions where they are installed. Among other exhibitors were the Abbey Effervescent Salt Company, who had an extensive display of their speciality. The Sanitas Company, Limited, who displayed their invaluable disinfectants, and Mr. H. W. Cox, who gave demonstrations with an X-ray apparatus which he exhibited. The Portia Company also had a stall which appeared to be of special interest to nurses.

